# Supplementary material for: MetaRibo-Seq measures translation in microbiomes
Source: Nat Commun. 2020 Jun 29;11:3268. doi: 10.1038/s41467-020-17081-z (PMC7324362; doi:10.1038/s41467-020-17081-z)
Supplement: Supplementary file 10 — Supplementary Data 7 [file 41467_2020_17081_MOESM10_ESM.zip › File2/Confidence_High_Taxonomy/319507_out.krona.html]

Javascript must be enabled to view this page.

members
magnitude
magnitudeUnassigned
count
unassigned
taxon
rank

319507\_out

262

2

SRS013476\_contig\_number\_contig-100\_1340.212256SRS015369\_contig\_number\_contig-100\_620.52896SRS019910\_contig\_number\_17900SRS024132\_contig\_number\_contig-100\_1275.268500SRS049959\_contig\_number\_13170
superkingdom
5
262

976
phylum
257

257
class
200643

257
171549
order

family
171552
2

1
genus
838

1262925
species

SRS049995\_contig\_number\_18831
1

1
genus
577309

1263095
species

SRS1055022\_contig\_number\_11114
1

1
2030927
species

SRS140513\_contig\_number\_8521

family
815
250

816

SRS011239\_contig\_number\_contig-100\_2411.154015SRS011302\_contig\_number\_30426SRS012849\_contig\_number\_34448SRS013098\_contig\_number\_contig-100\_476.291650SRS013158\_contig\_number\_5265SRS014287\_contig\_number\_contig-100\_190.116592SRS014613\_contig\_number\_21003SRS014683\_contig\_number\_18300SRS015190\_contig\_number\_16604SRS015854\_contig\_number\_63SRS017103\_contig\_number\_38850SRS017307\_contig\_number\_contig-100\_1447.172743SRS017916\_contig\_number\_15819SRS018049\_contig\_number\_contig-100\_283.284SRS018427\_contig\_number\_32177SRS019068\_contig\_number\_52064SRS020233\_contig\_number\_contig-100\_3339.284521SRS020328\_contig\_number\_34922SRS023583\_contig\_number\_16900SRS023971\_contig\_number\_2706SRS045004\_contig\_number\_5714SRS045713\_contig\_number\_20674SRS048870\_contig\_number\_17864SRS050801\_contig\_number\_3835SRS050974\_contig\_number\_39SRS053398\_contig\_number\_3549SRS054059\_contig\_number\_contig-100\_758.49461SRS054956\_contig\_number\_contig-100\_238.93672SRS064276\_contig\_number\_42243SRS064557\_contig\_number\_2194SRS077392\_contig\_number\_contig-100\_1601.230664SRS098655\_contig\_number\_contig-100\_135.73929SRS101433\_contig\_number\_contig-100\_706.95741SRS103987\_contig\_number\_35411SRS1041033\_contig\_number\_contig-100\_2958.140603SRS1041139\_contig\_number\_contig-100\_1147.1148SRS1055069\_contig\_number\_contig-100\_794.85635SRS1055076\_contig\_number\_13762SRS142980\_contig\_number\_687SRS143070\_contig\_number\_356SRS143722\_contig\_number\_contig-100\_34.89345SRS147039\_contig\_number\_contig-100\_77.26262SRS148424\_contig\_number\_29484SRS148721\_contig\_number\_55498SRS893292\_contig\_number\_8929SRS893358\_contig\_number\_14217SRS893366\_contig\_number\_26961SRS893369\_contig\_number\_contig-100\_1466.67152SRS893378\_contig\_number\_21183
49
250
genus

1
species

SRS021948\_contig\_number\_18439
291644

4
2184759

SRS013687\_contig\_number\_contig-100\_366.171947SRS023914\_contig\_number\_22034SRS101268\_contig\_number\_contig-100\_703.14622SRS1041037\_contig\_number\_6631
species

2

SRS014235\_contig\_number\_662SRS042284\_contig\_number\_contig-100\_84.48058
species
371601

1
674529

SRS045244\_contig\_number\_3331
species

species

SRS050941\_contig\_number\_3925
28116
1

species

SRS018836\_contig\_number\_11855SRS019601\_contig\_number\_30577
2292928
2

1
329854

SRS020622\_contig\_number\_7547
species

2
1946089

SRS014855\_contig\_number\_8335SRS147919\_contig\_number\_16505
species

2

SRS015890\_contig\_number\_6130SRS147346\_contig\_number\_4926
species
818

species

SRS063518\_contig\_number\_33326SRS1041164\_contig\_number\_6525SRS1055083\_contig\_number\_13065
2292927
3

species

SRS101376\_contig\_number\_1281
1263054
1

8

SRS013940\_contig\_number\_21068SRS014923\_contig\_number\_45859SRS015431\_contig\_number\_28236SRS050925\_contig\_number\_36857SRS077335\_contig\_number\_3225SRS1041091\_contig\_number\_16734SRS1041138\_contig\_number\_10570SRS893253\_contig\_number\_1793
species
93975

1
species

SRS893256\_contig\_number\_485
821

2
46506

SRS017859\_contig\_number\_2989SRS077552\_contig\_number\_720
species

820
species

SRS011134\_contig\_number\_42849SRS012969\_contig\_number\_41033SRS013521\_contig\_number\_9839SRS013951\_contig\_number\_38351SRS014948\_contig\_number\_15361SRS015065\_contig\_number\_8334SRS015579\_contig\_number\_5064SRS015663\_contig\_number\_36044SRS015694\_contig\_number\_23446SRS015794\_contig\_number\_11649SRS016056\_contig\_number\_5790SRS016840\_contig\_number\_2734SRS016954\_contig\_number\_26305SRS017433\_contig\_number\_12167SRS017521\_contig\_number\_15583SRS017701\_contig\_number\_6716SRS017745\_contig\_number\_2232SRS018541\_contig\_number\_854SRS018575\_contig\_number\_8386SRS018936\_contig\_number\_2160SRS018984\_contig\_number\_14232SRS019397\_contig\_number\_8089SRS019445\_contig\_number\_15320SRS019685\_contig\_number\_35386SRS019693\_contig\_number\_444SRS019808\_contig\_number\_5736SRS020394\_contig\_number\_9422SRS021153\_contig\_number\_2226SRS021219\_contig\_number\_12337SRS021484\_contig\_number\_20060SRS022137\_contig\_number\_19784SRS022524\_contig\_number\_13557SRS022609\_contig\_number\_51558SRS022713\_contig\_number\_5408SRS023526\_contig\_number\_contig-100\_54.229707SRS024009\_contig\_number\_1498SRS024331\_contig\_number\_37457SRS024625\_contig\_number\_7573SRS042966\_contig\_number\_20431SRS043411\_contig\_number\_16529SRS043768\_contig\_number\_14350SRS045826\_contig\_number\_16661SRS046502\_contig\_number\_9629SRS046717\_contig\_number\_1250SRS048060\_contig\_number\_1582SRS048164\_contig\_number\_32694SRS049402\_contig\_number\_13964SRS049896\_contig\_number\_31789SRS050026\_contig\_number\_12138SRS050422\_contig\_number\_37854SRS050520\_contig\_number\_216SRS051031\_contig\_number\_12395SRS051610\_contig\_number\_8197SRS052078\_contig\_number\_1319SRS052697\_contig\_number\_1418SRS053087\_contig\_number\_1310SRS053649\_contig\_number\_19483SRS055017\_contig\_number\_73SRS055982\_contig\_number\_18203SRS057478\_contig\_number\_6239SRS058723\_contig\_number\_contig-100\_49.63284SRS062427\_contig\_number\_9911SRS062701\_contig\_number\_5403SRS063370\_contig\_number\_4711SRS063489\_contig\_number\_7880SRS064973\_contig\_number\_2680SRS075716\_contig\_number\_2987SRS075821\_contig\_number\_856SRS076756\_contig\_number\_11716SRS076804\_contig\_number\_16952SRS076976\_contig\_number\_5493SRS077024\_contig\_number\_12495SRS077589\_contig\_number\_574SRS077641\_contig\_number\_contig-100\_315.126123SRS077849\_contig\_number\_22301SRS078242\_contig\_number\_30093SRS078419\_contig\_number\_24749SRS078665\_contig\_number\_10360SRS098061\_contig\_number\_9169SRS098717\_contig\_number\_23186SRS104084\_contig\_number\_5097SRS1041039\_contig\_number\_27553SRS1041116\_contig\_number\_8705SRS1041130\_contig\_number\_5018SRS1041133\_contig\_number\_27849SRS1041137\_contig\_number\_16068SRS1041142\_contig\_number\_4527SRS1041143\_contig\_number\_6940SRS1041145\_contig\_number\_34091SRS104400\_contig\_number\_13928SRS104975\_contig\_number\_24111SRS1054691\_contig\_number\_contig-100\_19.111148SRS1055038\_contig\_number\_5025SRS1055043\_contig\_number\_24057SRS1055049\_contig\_number\_16163SRS1055067\_contig\_number\_16531SRS1055095\_contig\_number\_504SRS1055099\_contig\_number\_15479SRS142503\_contig\_number\_755SRS142542\_contig\_number\_24717SRS142781\_contig\_number\_266SRS142923\_contig\_number\_15968SRS143181\_contig\_number\_4341SRS143372\_contig\_number\_7636SRS143466\_contig\_number\_18121SRS143991\_contig\_number\_28594SRS144183\_contig\_number\_29812SRS144297\_contig\_number\_contig-100\_49.106920SRS144603\_contig\_number\_12584SRS144714\_contig\_number\_29339SRS145497\_contig\_number\_1488SRS147271\_contig\_number\_14158SRS147377\_contig\_number\_7528SRS147653\_contig\_number\_836SRS147766\_contig\_number\_6382SRS148817\_contig\_number\_12399SRS149075\_contig\_number\_11673SRS149244\_contig\_number\_13168SRS892882\_contig\_number\_14551SRS893172\_contig\_number\_451SRS893187\_contig\_number\_737SRS893231\_contig\_number\_5236SRS893295\_contig\_number\_12340SRS893300\_contig\_number\_11535SRS893373\_contig\_number\_3883SRS893380\_contig\_number\_2953
126

817
species

SRS140492\_contig\_number\_16805SRS971275\_contig\_number\_16437
2

3
47678

SRS077294\_contig\_number\_4885SRS149784\_contig\_number\_18311SRS893252\_contig\_number\_11118
species


SRS098571\_contig\_number\_65888
species
2292931
1

1739298

SRS143523\_contig\_number\_14460
species
1

310298

SRS016989\_contig\_number\_13874SRS019030\_contig\_number\_15680SRS019161\_contig\_number\_38415SRS019496\_contig\_number\_14978SRS045645\_contig\_number\_4736SRS052027\_contig\_number\_15200SRS054905\_contig\_number\_19675SRS064232\_contig\_number\_2891SRS065504\_contig\_number\_15454SRS077194\_contig\_number\_12791SRS077454\_contig\_number\_13055SRS078176\_contig\_number\_22518SRS1041032\_contig\_number\_15791SRS104311\_contig\_number\_22736SRS104636\_contig\_number\_29046SRS104693\_contig\_number\_11530SRS144537\_contig\_number\_49295SRS147088\_contig\_number\_contig-100\_941.43201SRS147139\_contig\_number\_18805SRS147977\_contig\_number\_7391SRS148196\_contig\_number\_21267SRS149879\_contig\_number\_24529SRS893279\_contig\_number\_7464SRS893342\_contig\_number\_4961SRS971276\_contig\_number\_20347
species
25

291645

SRS1041134\_contig\_number\_457
species
1

457387
species

SRS075984\_contig\_number\_13306
1

1

SRS893383\_contig\_number\_22832
species
2292941

1
457395
species

SRS014313\_contig\_number\_4161

3
species

SRS011405\_contig\_number\_9571SRS053335\_contig\_number\_10655SRS150029\_contig\_number\_7788
585543

1
species

SRS019286\_contig\_number\_3201
2292913

2
2292924

SRS020271\_contig\_number\_2215SRS056259\_contig\_number\_25385
species

species

SRS014979\_contig\_number\_23636SRS016203\_contig\_number\_2066
246787
2

1
171550
family

239759
genus
1

28117
species

SRS065176\_contig\_number\_3578
1

family
1853231
1

1
283168
genus

1
species

SRS011084\_contig\_number\_27867
28118

2005525
family
2

genus
375288
2


SRS015960\_contig\_number\_13428SRS075878\_contig\_number\_18405
species
387661
2
